# How bacteria use electric fields to reach surfaces

**DOI:** 10.1016/j.bioflm.2021.100048

**Published:** 2021-04-08

**Authors:** Poehere Chong, Benjamin Erable, Alain Bergel

**Affiliations:** Laboratoire de Génie Chimique, Université de Toulouse, CNRS, INP, UPS, Toulouse, France

**Keywords:** Electroactive biofilms, Electrotaxis, Galvanotaxis, Microbial fuel cell, Microbial electrochemical technology, Bioelectrochemical system

## Abstract

Electrotaxis is the property of cells to sense electric fields and use them to orient their displacement. This property has been widely investigated with eukaryotic cells but it remains unclear whether or not bacterial cells can sense an electric field. Here, a specific experimental set-up was designed to form microbial electroactive biofilms while differentiating the effect of the electric field from that of the polarised electrode surface. Application of an electric field during exposure of the electrodes to the inoculum was shown to be required for an electroactive biofilm to form afterwards. Similar biofilms were formed in both directions of the electric field. This result is attributed to the capacity of the cells to detect the K^+^ and Na^+^ ion gradients that the electric field creates at the electrode surface. This microbial property should now be considered as a key factor in the formation of electroactive biofilms and possible implications in the biomedical domain are discussed.

## 1 Introduction

It is becoming increasingly obvious that microbial biofilms have a natural tendency to exchange electrons with their support when they grow on a conductive surface [1]. For around two decades, microbial electroactive biofilms have been the source of the so-called microbial electrochemical technologies [[2], [3], [4]] with a huge number of possible application fields such as the production of electrical energy [5,6] or hydrogen [7], effluent treatments [[8], [9], [10]] and metal recovery [11] for example. Electroactive biofilms have also essential roles in microbiologically influenced corrosion [12]. Beside the field of conventional electrochemical processes, more and more cases of inter-species microbial electron transfers are being discovered to be mediated by conductive solids [[13], [14], [15]].

Extensive fundamental research has led to great advances in our understanding of extracellular electron transfers inside biofilms and between biofilms and electrodes [[16], [17], [18]]. Nevertheless, we still know very little about the early formation of electroactive biofilms. In particular, the way the microbial cells approach a solid surface in the presence of an electric field remains unclear. It seems tacitly accepted that microbial cells reach the surface of polarised electrodes by random motion and that the electrode impacts the biofilm development only during the phase of cell growth on the surface. The possible impact of the electric field on the approach phase, before the cells reach the electrode surface, is rarely evoked.

A few studies have proposed that bacterial cells can migrate in the electric field as colloids that are uniformly charged [19] or behave as dipoles [20]. In these cases, bacterial cells have been assumed to migrate passively, without the involvement of a specific sensing process. In contrast, a few reports have postulated that bacteria might detect local electric fields [21] through chemotaxis, by sensing the concentration gradient of redox compounds [22,23]. Some reports have described the inhibition of cell motility by an applied current, without evoking possible mechanisms [24,25]. Others have observed that the swimming speed of *Shewanella* species increases in the vicinity of a polarised electrode [26]. This effect is clearly different from a passive migration process because the swimming speed was enhanced in all directions of motion rather than in the direction of the electric field only. Nevertheless, the effect was conditioned by the cells’ ability to exchange electrons with the electrode and therefore required an initial contact with the electrode to be triggered. Apart from these few leads, the approach phase of bacterial cells towards a polarised surface is still very poorly documented.

In contrast, eukaryotic cells have been widely demonstrated to develop sophisticated strategies to sense an electric field and use it to orient their motion [27,28]. This property, called electrotaxis, has been shown to play key roles in essential physiological processes such as tissue development [29], wound healing [30], and organ formation [31,32]. The multiplicity of evidence of electrotaxis in eukaryotic cells prompted us to look for a possible bacterial electrotaxis-related strategy in the formation of electroactive biofilms.

The purpose of this study was to move forward in identifying and deciphering possible microbial electrotaxis by using a complex multi-species consortium. A new type of electrochemical set-up was designed in order to discriminate the impact of the electric field from that of the polarised electrode. Actually, imposing an electric field through a solution requires electrochemical reactions to occur on the surface of the electrodes. It is consequently difficult to discriminate the impact of the electric field from that of the polarised electrode surface in a conventional electrochemical reactor, because the electrodes that create the electric field act also as the electron source or sink for the electroactive species that reach them. The new experimental device and procedure described here made it possible to separate the two effects.

It is thus evidenced here that an electric field enhances the formation of electroactive biofilms on solid surfaces. The electric field affects the biofilm formation through indirect action due to the ionic gradient created at the interface. This analysis leads us to consider the capacity of microbial cells to detect interfacial ionic gradients as a major motor of surface colonisation.

The results described here indicate that controlling local ionic gradients may be an efficient way to enhance or mitigate the formation of biofilms on solid surfaces. Obvious applications can be foreseen in all domains of microbial electrochemical technologies. Beyond that, and in a more speculative way, considering the ubiquitous presence of endogenous electric fields in living organisms [30] and the huge number of interfaces that compose them, the results described here may also open up new research paths in the domains of biomedicine and physiology.

## 2 Materials and methods

### 2.1 Inoculum and media

The inoculum solution was garden compost leachate prepared by filtering a mix of 1.5 ​L of garden compost (Lombricompost, Or Brun) and 2.25 ​L of tap water containing 60 ​mM KCl through a large-mesh cloth. The synthetic medium was a phosphate buffer solution supplemented or not with 60 ​mM of KCl. The phosphate buffer solution was obtained by mixing a solution of Na_2_HPO_4_ (9.47 ​g ​L^-1^) and a solution of KH_2_PO_4_ (9.08 ​g ​L^-1^) with a ratio of respectively 61-39% (v/v) to a final pH of 7.0. When indicated in the protocol, the synthetic medium was supplemented with 20 ​mM of sodium acetate and with 10 ​mL ​L^-1^ of macronutrients solution, 1 ​mL ​L^-1^ of micronutrients solution and 1 ​mL ​L^-1^ of vitamins solution (see Supplementary Information Table S1).

### 2.2 Electrochemical set-up (Fig. 1)

The electrochemical reactors were made by assembling three modified Schott glass bottles (diameter 101 ​mm, height 152 ​mm, Duran). The central compartment was separated from the two end compartments with a system constituted of a flat gasket cut from a rubber foil with an outer diameter of 5 ​cm and an inner diameter of 2 ​cm, a membrane with 0.2-μm cut-off threshold (outer diameter 5 ​cm, Pall SAS France), a carbon cloth electrode (outer diameter 3 ​cm, PaxiTech SAS France) electrically connected with a platinum wire, and a second rubber gasket identical to the first one. Such a separation system was arranged between the central compartment and each of the two end compartments, with the carbon cloth electrode facing the central compartment. According to the inner diameter of the flat rubber gasket (2 ​cm), the surface area of the carbon cloth electrodes exposed to the solution was of 3.1 ​cm^2^. Sealing was achieved by pressing the system with an external frame equipped with four bolts.

**Fig. 1:**
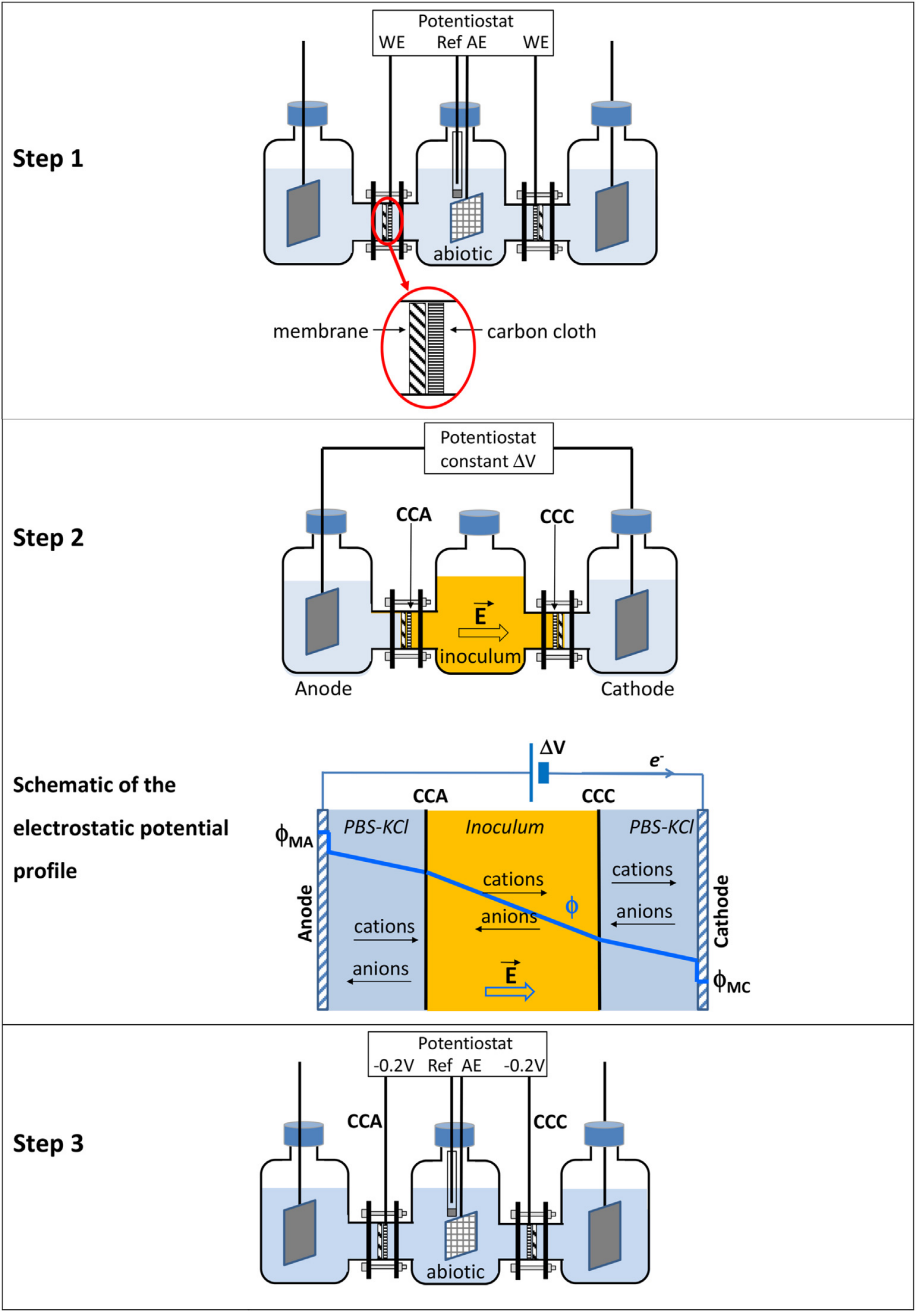
Scheme of the experimental set-up and protocol. The carbon cloth electrodes (CCA and CCC) were in the central compartment, applied against the membrane surfaces. A dimensionally stable anode and a stainless steel cathode were located in the anode and cathode end compartments, respectively. **Step 1** A platinum grid (auxiliary electrode AE) and a saturated calomel electrode (SCE, Ref) were introduced into the central compartment. CCA and CCC were connected individually as working electrodes (WE) to be electrochemically pre-treated in a synthetic medium. **Step 2** The auxiliary and reference electrodes were removed from the central compartment, which was then filled with deoxygenated compost leachate, under anaerobic conditions. An electric field was achieved by maintaining 8 V voltage (ΔV) between the anode and the cathode contained in the end compartments. Nitrogen was continuously bubbled into the end compartments. The scheme describes the profile of the electrostatic potential ϕ. **Step 3** The compost leachate was removed from the central compartment under anaerobic conditions and replaced by deoxygenated phosphate buffer solution (PBS) that could contain KCl or not. The auxiliary and reference electrodes were put into the central compartment. CCA and CCC were individually connected as working electrodes and polarised at -0.20 V vs. SCE.

A dimensionally stable anode (DSA) of 8 ​cm ​× ​2 ​cm x 0.1 ​cm was placed in an end compartment and a stainless steel electrode (SS) of 3 ​cm ​× ​3 ​cm x 0.3 ​cm was placed in the other end compartment. The stainless steel electrode was cleaned with a 50-50% (v/v) mixture of acetone-ethanol, then with a 2–20% fluoronitric acid solution and finally rinsed with distilled water. These two metallic electrodes were electrically connected with a 2-mm-diameter screwed titanium wire. The electric field was established between these two metallic electrodes by applying a voltage of 8 ​V during 3 ​h (or 2 ​h when indicated) between the DSA used as the anode and the SS electrode used as the cathode, which were separated by around 27 ​cm.

When necessary, a saturated calomel reference electrode (SCE, Radiometer, +0.241 ​V vs SHE) and a platinum grid used as the counter electrode were introduced in the central compartment and each of the two carbon cloth electrodes was individually polarised as a working electrode at -0.20 ​V/SCE with a multi-channel potentiostat (N-stat set-up, Biologic SA, France).

### 2.3 Experimental procedure (Fig. 1)

Step 1 *Electrochemical pre-treatment of the carbon cloth electrodes.* The two carbon cloth electrodes were submitted to an electrochemical pre-treatment in order to ensure that both had the same initial surface state before exposure to the inoculum. The three compartments were filled with 700 ​mL of synthetic medium. A reference electrode (SCE) and a platinum grid used as the counter (CE) electrode were introduced in the central compartment and the two carbon cloth electrodes, individually addressed as working electrodes, were polarised at -0.20 ​V/SCE with an N-stat device. After 35 ​min of polarisation, cyclic voltammetry was recorded with each carbon cloth electrode at 1 ​mV/s in the -0.5 to +0.2 ​V*/*SCE range to check their identical initial electrochemical characteristics.
Step 2 *Inoculating with compost leachate and imposing an electric field.* The SCE and the CE electrodes were removed from the central compartment. The medium was removed from the central compartment and replaced by garden compost leachate by using a pump and keeping the compartment under constant nitrogen flow during the operation. Compost leachate had been previously deoxygenated by 30 ​min of vigorous nitrogen bubbling. Then, 20 ​mM of sodium acetate was added in the three compartments. During 3 ​h, a voltage of 8 ​V was applied between the anode (DSA) and the cathode (SS). The carbon cloth electrodes were not connected to an electrical circuit during this step. Nitrogen was continuously bubbling in the end compartments. Based on this standard procedure, a few parameters have been varied (Table 1). For some experiments a synthetic medium that didn’t contain KCl was used in the end compartments, or the exposure time to the electric field was reduced to 2 ​h.

**Table 1:** Starting time and maximum current density (Max J) observed in Step 3 (from Figs. 2 and S1). The first two columns give the operating conditions used in Step 2. The starting time was arbitrarily defined as the time at which current density reached 0.24 A.m^-2^.

*Step 2*		*Step 3*			
Electric field	KCl in end compartments	CCA		CCC	
		*Starting time (day)*	*Max J (A.m*^*-2*^*)*	*Starting time (day)*	*Max J (A.m*^*-2*^*)*
no	yes	6.1	0.37	–	0.07
		–	0.03	–	0.03
2 ​h	yes	6.9	0.46	6.3	2.9
		6.1	0.37	–	0.19
3 ​h	yes	5.1	9.0	3.7	11.6
		6.3	3.9	4.3	10.6
3 ​h	no	4.8	3.6	4.4	3.4
		5.3	0.24	4.4	0.5
Step 3 *Polarisation of the carbon cloth electrodes in the absence of the inoculum.* The compost leachate was removed from the central compartment and replaced by 700 ​mL of synthetic medium supplemented with 20 ​mM sodium acetate, always under constant nitrogen flux. The medium was previously deoxygenated by 30 ​min of vigorous nitrogen bubbling. Macronutrients, micronutrients and vitamins were added into the three compartments. The reference and counter electrodes were placed again into the central compartment. The two carbon cloth electrodes were individually polarised at -0.20 ​V/SCE during 7 days. At day 6, the concentration of sodium acetate was checked in all compartments with an enzymatic kit (K-ACETAK, Megazymes) and adjusted at 20 ​mM. At the end of the polarisation, CV curves were recorded at low scan rate (1 ​mV/s) starting from the polarisation value (-0.20 ​V/SCE) and in the range from -0.5 to +0.2 ​V*/*SCE.

All experiments were carried out in a heat chamber at 40 ​°C. A previous study has shown that this temperature value was optimal to accelerate the formation of electroactive biofilms from this inoculum, even if the biofilm is then intended to work at room temperature [33]. This pretty high value should be due to the origin of the inoculum, derived from compost, and therefore developed at relatively high temperature. The two end compartments were continuously flushed with nitrogen from Step 1 to 3. The central compartment was only deaerated by 15 ​min nitrogen bubbling at the beginning of each step and nitrogen was continuously flowing through it during solution replacements. Current densities and molar fluxes were related to the projected surface area of the carbon cloth electrodes exposed to the solution (3.14 ​cm^2^).

### 2.4 Characterization of the carbon cloth electrodes

At the end of the experiments, each carbon cloth electrode was removed from the reactor and divided into three pieces to be imaged by epifluorescence microscopy and scanning electron microscopy and for analysis of the microbial population by16S rRNA pyrosequencing.

*Epifluorescence microscopy.* Samples were stained with acridine orange 0.01% (A6014 Sigma) during 10 ​min, then rinsed in water and dried at ambient temperature overnight. Staining with acridine orange marked both extracellular and intracellular nucleic acids so that imaging gave a fair representation of the global biofilm coverage. The samples were imaged with a Carl Zeiss Axiomalger M2 microscope equipped for epifluorescence with an HBO 50 ​W ac mercury light source and the Zeiss 09 filter (excitor HP450-490, reflector FT 10, Barrier filter LP520). Images were acquired with a monochrome digital camera (evolution VF) every 3.9 ​μm along the Z-axis and the set of images was processed with the Axiovision® software. Four different spots were imaged for each electrode to acquire a representative vision of biofilm coverage.

*Scanning electron microscopy.* Samples were fixed in phosphate buffer solution (0.4 ​M, pH 7.4) containing 4% of glutaraldehyde. They were then washed with phosphate buffer and saccharose (0.4 ​M) and dehydrated by immersion in increasing concentrations of acetone (50%, 70%, 100%), then in a mixture of acetone and hexamethyldisilazane (HMDS) (50:50), and finally in 100% HMDS. The last batch of HMDS was dried until complete evaporation. Samples were observed with a scanning electron microscope LEO 435 VP. Four different spots were imaged for each electrode to acquire a representative vision of the biofilm structure.

*Microbial community analysis.* Biofilms were detached from the carbon cloth electrodes in 15 ​mL of sterile phosphate buffer solution by sonication (30 ​min, 80 ​W). Samples of 20 ​mL were also collected from the garden compost leachate (inoculum). For all samples (from the electrodes and the inoculum solution), cells were concentrated by centrifugation at 4700 ​rpm during 8 ​min at 10 ​°C. DNA was extracted using the MOBIO PowerSoil ® DNA Isolation kit according to the manufacturer’s manual. A 28F-519R bacterial 16s assay was performed on the MiSeq system to identify the microbial communities (RTLGe-nomics, Lubbock, USA).

## 3 Results and discussion

### 3.1 Experimental set-up and protocol

The electric field propagates between two polarised electrodes by the transport of ionic species through the solution. It may thus have a great impact on the approach of bacterial species to the electrode, particularly during biofilm formation. In a conventional electrochemical reactor, it is very difficult to distinguish the effect of the electric field, which acts in solution, from the effect of contact with the polarised surface, because bacterial cells are influenced by the polarised surface as soon as they come into contact with it. It was consequently necessary to design a new type of electrochemical set-up in order to discriminate between the effect of the electric field and that of the polarised surface.

The core of the experimental set-up was composed of two carbon cloth electrodes placed face to face at the extremities of a central compartment. The electric field was imposed by external electrodes, which were located beyond the carbon cloth electrodes, in the end compartments (Fig. 1). The central compartment and the end compartments were separated by semipermeable membranes, against which the carbon cloth electrodes were held. The experimental protocol was organised in three steps.

The first step was dedicated to electrode pre-treatment because it was important to start the experiment with the two electrodes (CCA and CCC) exhibiting identical surface state. The pre-treatment, which consisted in 35 ​min polarisation at -0.2 ​V/SCE, was considered effective as it led to similar voltammetric records for both CC electrodes (Fig. S1 in Supplementary data). If the voltammetric records were not identical for CCA and CCC, the experiment would be stopped and the electrode changed.

Then, during Step 2, the central compartment was filled with the inoculum solution and the carbon cloth electrodes were exposed to the inoculum for 3 ​h, while the cell voltage *ΔV* of 8 ​V was applied between the external electrodes. During this exposure step, the carbon cloth electrodes were not connected to the electrical circuit. One carbon cloth electrode was closer to the external anode (CCA), the other closer to the external cathode (CCC). Control experiments were performed without applying the voltage.

During Step 2, when the voltage ΔV was applied, it imposed a difference of the same value to the electrostatic potential of the two external electrodes (Schematic in Fig. 1):

(1) ΔV ​= ​ϕ_MA_ - ϕ_MC_

where ϕ_MA_ and ϕ_MC_ are the electrostatic potential of the anode and cathode materials, respectively. A gradient of electrostatic potential was thus driven through the whole system. This gradient established an electric field in the whole system and drove the motion of charged species, which transported the current through the bulk. Basics and equations that relate these different parameters, including the relationship between electrostatic potential and Nernst potential, have been detailed in a recent article [34].

Finally, during Step 3, the inoculum solution was removed from the central compartment and replaced by a synthetic medium. The two carbon cloth electrodes were connected to the electrical circuit and both polarised at -0.20 ​V/SCE for 7 days. The objective of Step 3 was to check whether an electroactive biofilm could form from the microbial species that had adhered to the electrode surface during Step 2. This step of polarisation in synthetic medium can be regarded as a kind of photographic development (for readers old enough to remember silver photography) revealing what had happened during the prior exposure to the inoculum. Garden compost was chosen as the inoculum because it has proved its capacity to form efficient microbial anodes [35,36].

### 3.2 Development of the electroactive biofilm

**Fig. 2:**
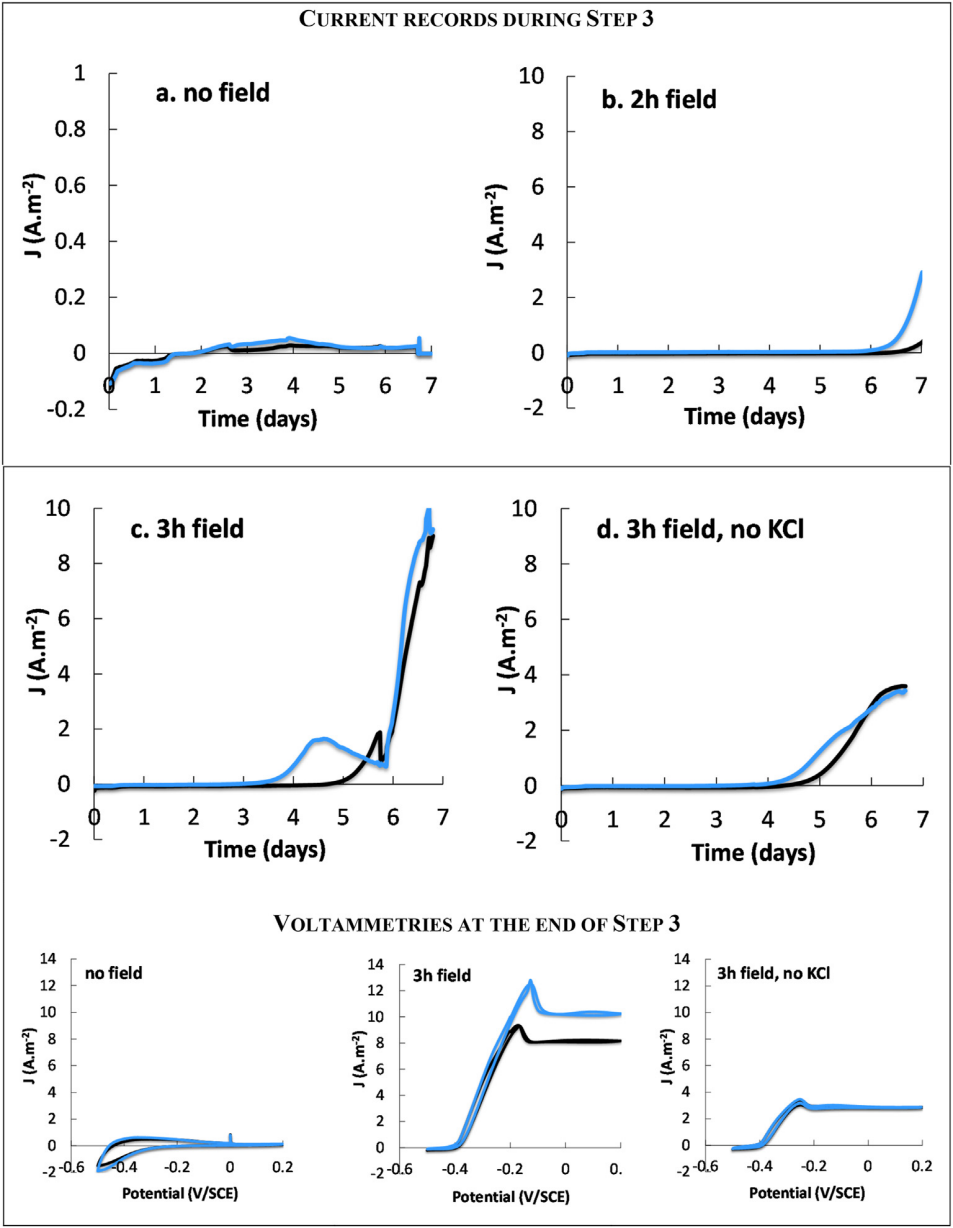
Currents recorded during the 7-day biofilm development and cyclic voltammetries performed on the 7-day biofilms (Step 3). Duplicates are reported in Fig. S2 in the Supplementary data. During Step 3, the CCC (blue lines) and CCA (black lines) electrodes were identically polarised at -0.20 ​V vs. SCE for 7 days. No current was produced when no electric field had been applied during Step 2 (records a). A small current density was obtained when the electric field was applied for only 2 ​h in Step 2 (records b). In contrast, considerable current density was observed when the electric field was applied for 3 ​h (records c and d), with lower values when there was no KCl in the end compartment (records d). Note that the y-axis scale of b, c and d was one order of magnitude higher than in a. (For interpretation of the references to colour in this figure legend, the reader is referred to the Web version of this article.) Cyclic voltammetry performed at the end of the 7-day polarisation period confirmed the common characteristic of an electroactive biofilm when the 3-h electric field had been applied. CCC electrodes started to produce current sooner than CCA electrodes and/or produced higher current density. No signal was displayed when the electric field had not been applied.

If microbial species able to develop electroactivity adhere to the carbon cloth electrodes during Step 2, an electroactive biofilm will form under the effect of the applied potential during Step 3. The formation of this biofilm will result in an increasing current intensity being recorded. Control experiments performed without applying the electric field during the exposure-to-inoculum step (Step 2) did not lead to the production of significant current during Step 3 (Fig. 2). At the end of the 7-day polarisation, cyclic voltammetries (CV) confirmed that no electroactive biofilm had formed on the control electrodes. SEM imaging showed no obvious biofilm structure (Fig. S3 in Supplementary data); only a few traces of salt crystals were observed and rare bacteria were detected at high magnification. Epifluorescence imaging confirmed that only a few isolated spots of the electrode surfaces were colonised (Fig. S4 in Supplementary data).

When the electric field was applied during Step 2, current was produced by both CCA and CCC electrodes during Step 3 (Fig. 2). The current densities were significantly higher when 60 ​mM KCl was present in the end compartments, and they were considerably lower when the electric field was applied for only 2 ​h instead of 3 during Step 2. The current always started first on the CCC electrodes. The lead of the CCC curves was greater when KCl was present in the end compartments and it was smaller when the electric field was applied for only 2 ​h (Table 1). CVs at the end of the 7 days of polarisation confirmed the presence of efficient electroactive biofilms, with current-potential curves similar to those already reported with garden compost inoculum [37,38]. CVs also confirmed the higher efficiency of the CCC electrode when KCl was present in the end compartments, while both CCA and CCC gave comparable characteristics in the absence of KCl. SEM showed scattered bacterial colonies on CCA electrodes, and a visible coating on CCC electrodes, which was greater when KCl was present in the end compartments (Fig. S3 in Supplementary data). At higher magnification, microbial aggregates and a few salt crystals were observed on the CCA electrodes, while CCC electrodes exhibited noticeable salt precipitation. However, epifluorescence indicated significant microbial colonisation on both CCC and CCA electrodes, with no apparent difference (Fig. S4 in Supplementary data). On both CCA and CCC electrodes, microbial colonisation was more marked when KCl was present in the end compartments.

To sum up, when no electric field was applied during exposure of the electrodes to the inoculum, the current recorded during the polarisation was almost nil and only a few scattered bacteria were observed on the electrodes after 7 days of polarisation. In contrast, when the electric field was applied during exposure to the inoculum, the electrodes started producing current after 3–4 days of polarisation. The current rapidly exceeded 1 A.m^-2^ and considerable surface colonisation was observed after the 7-day polarisation. These experiments showed that the presence of an electric field was necessary during exposure to the inoculum if the bacteria were to reach the electrode surface in sufficient quantity to subsequently form a biofilm during polarisation. It can thus be concluded that the electric field plays an essential role in the pioneering settlement of a surface by electroactive bacteria.

### 3.3 Similar microbial communities

Surprisingly, the electrodes positioned downstream of the electric field (CCC), i.e. close to the cathode that established the electric field, started to produce current first and often produced higher current density than the CCA electrodes (Table 1). Most bacterial cells bear an overall negative charge on their external wall at pH above around 4 [39]. Out of an electric field, these charges are shielded by cations coming from the solution and the cells display zero global charge. In the presence of an electric field, some of the cations are pulled away from the cell surface and the cells recover a negative charge. It was therefore expected that most bacterial cells would migrate towards the anode. Accordingly, here, the CCA electrodes should have received a higher inflow of bacterial cells, while CCC colonisation would have been hindered. However, the experimental data indicated the opposite: CCC electrodes showed shorter response time and higher maximum current density than CCA electrodes. It must be concluded that simple migration of microbial cells under an electric field cannot explain the results observed here.

It might be argued that some microbial cells have positive surface charge [40], so the CCC electrodes could have been colonised by positively-charged cells while the CCA were colonised by the more common, negatively charged ones. This argument is invalidated by the analysis of the microbial communities that composed the biofilms.

At the end of the 7 days of polarisation, the amounts of DNA collected from the electrodes that had not been submitted to the electric field were not sufficient for pyrosequencing, which was consistent with the poor colonisation observed by epifluorescence and the very small current densities recorded. Consequently, only the microbial composition of the CCA and CCC electrodes obtained after exposure to the electric field were analysed and compared to that of the compost leachate used as the inoculum.

At the phylum level, the inoculum was composed of 33% of *Proteobacteria*, 23% of *Firmicutes*, 12% of *Actinobacteria*, 14% of other minor phyla, and 18% of the total reads were not identified. In comparison, the microbial communities of the carbon cloth electrodes (both CCA and CCC) showed clear enrichment in *Proteobacteria*, whose relative abundance ranged from 79 to 91%. A few sequences belonged to the phylum *Firmicutes* (7–17%). The selective enrichment of *Proteobacteria* has commonly been observed in electroactive biofilms as several bacteria known to be electroactive (e.g. *Geobacter* and *Shewanella*) belong to this phylum. *Firmicutes* is also known to host electroactive species [41].

**Fig. 3:**
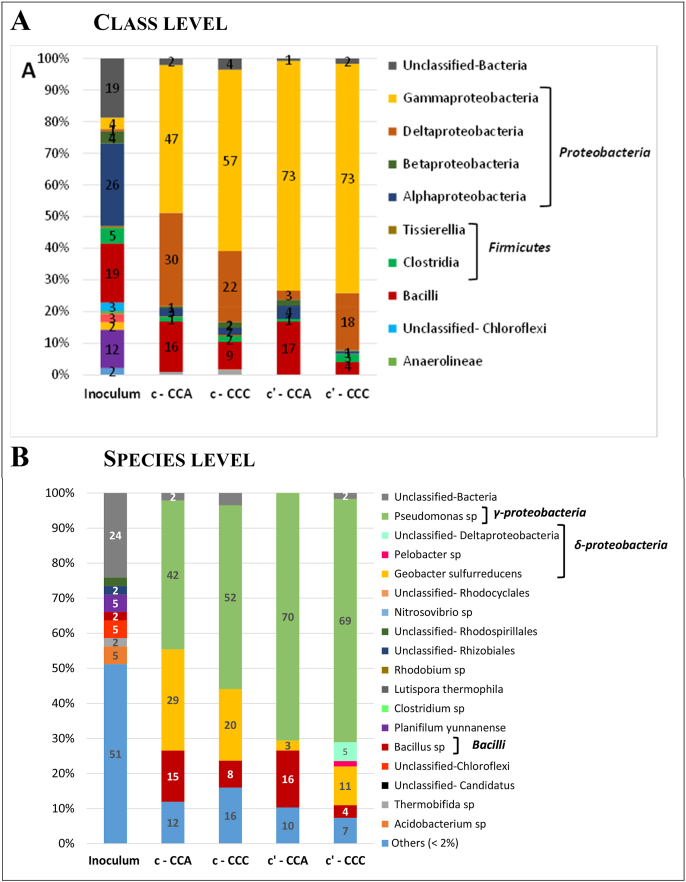
Relative abundances of major A) class and B) species in the inoculum and CCA and CCC electrodes. The four carbon cloth electrodes were exposed to the 3-h electric field during Step 2 with KCl in the end compartments (records c and c’ in Fig. 1, Fig. 2, respectively). Relative abundances of species inferior to 2% were defined as “Others”. The biofilms of both CCA and CCC electrodes showed similar selection of the same three species: Pseudomonas sp (Gamma-proteobacteria), Geobacter sulfurreducens (Delta-proteobacteria) and Bacillus sp (Bacilli), which always constituted more than 80% of the community. There was no marked difference in the relative abundance of these three dominant species between CCC and CCA electrodes.

At the class level (Fig. 3A) the *Proteobacteria* phylum included a majority of *Alpha-proteobacteria* (26%) for the inoculum, while it was mostly divided between *Gamma-* (from 47 to 73%) and *Delta-proteobacteria* (from 3 to 30%) on the electrodes. *Bacilli* emerged as the dominant class of the *Firmicutes* phylum in both the inoculum (19%) and the electrodes (from 4 to 17%). The same classes were present on CCA and CCC electrodes.

At the species level (Fig. 3B), the inoculum showed a wide variety. More than half (51%) of community was composed by species that had a relative abundance of less than 2%. A total of 7 species were identified and detected at relative abundances between 2 and 5%. Twenty-four percent of the total microbial population was not identified.

The microbial population variety was considerably more restricted on the electrode surfaces. 95% of the total population was identified and more than 80% was covered by only 3 species: *Pseudomonas* sp *(Gamma-proteobacteria), Geobacter sulfurreducens (Delta-proteobacteria)* and *Bacillus* sp *(Bacilli)*. *Geobacter sulfurreducens* is one of the most widely reported electroactive microorganisms [[42], [43], [44], [45]]. It was not possible to identify the species of *Pseudomonas* and *Bacillus* present on the electrode surfaces. Nevertheless, many species of *Pseudomonas* have been reported as electroactive, such as *Pseudomonas aeruginosa* [46,47], *Pseudomonas alcaliphila* [48,49]*,* and *Pseudomonas fluorescens* [47,50]. Some bacillus species have also been shown to be electroactive*,* e.g. *Bacillus subtilis* [47,51].

In conclusion, the biofilms of both CCA and CCC electrodes showed similar selection of the same three species: *Pseudomonas* sp *(Gamma-proteobacteria*), *Geobacter sulfurreducens* (*Delta-proteobacteria*) and *Bacillus* sp (*Bacilli*), which always made up more than 80% of the community and are related to species known for their electroactivity. There was no marked difference in the relative abundance of these three dominant species between CCC and CCA electrodes, so the hypothesis of biofilm formation by different species on CCC and CCA cannot be supported.

### 3.4 Interfacial ion fluxes

The effect of the electric field was significantly lower in two cases: when it was applied for only 2 ​h instead of 3 ​h, and when KCl was not present in the end compartments. The ionic current density (J_field_) that was forced through the carbon cloth electrodes during the application of the electric field was much higher (81.7 ​± ​23.4 A.m^-2^) when KCl was present in the end compartments than when it was absent (23.5 ​± ​0.7 A.m^-2^). In the presence of KCl, the end compartments had a lower ionic resistance. Consequently the current flowing through the reactor was drastically increased, according to Ohm’s law:

(2) *ΔV* = (*R*_*ec*_ ​+ ​*R*_*cc*_ ​+ ​*R*_*ec*_) *I*_*field*_

where *ΔV* is the applied voltage, *R*_*ec*_ and *R*_*cc*_ are the ionic resistances of the end compartments and the central compartment, respectively and *I*_*field*_ (A) is the intensity of the current that flows through the solution.

The value of the voltage *ΔV* was always 8 ​V, but the presence of KCl in the end compartments decreased *Rec* and thus increased *I*_*field*_*.* The dependence of the impact of the electric field on the presence or absence of KCl in the end compartments led us to believe that exposure to the inoculum was impacted by the ionic flux flowing through the carbon cloth electrodes.

The ionic fluxes induced by the electric field through the two solutions can be calculated by using the transport number (*t*_*i*_) of each ion, *i,* which is expressed as [52]:

(3) $$t_{i}=\cdot \frac{\lambda_{i}^{0}C_{i}}{\sum_{k}\lambda_{k}^{0}C_{k}}$$

where *λ*^*0*^ are the molar ionic conductivities (m^2^].S.mol^-1^) and *C* are the concentrations (mol.m^-3^). The molar flux density of each ion (*Φ*_*i*_ mole.s^-1^.m^-2^) is directly derived from the transport number as:

(4) $$\Phi_{i}\cdot =\cdot \frac{t_{i}}{z_{i}}\frac{J_{field}}{F}$$

where *z*_*i*_ is the charge of the species, *J*_*field*_ (A.m^-2^) is the current density that flows through the solution, and *F* is the Faraday constant (96 ​485C ​mol^-1^).

The transport number, *t*_*i*_, gives the proportion of charge that is transported through the solution by each ion. Similarly, according to Equation (4), the ratio $\frac{t_{i}}{z_{i}}$ gives the proportion of the total ionic flux ensured by each ion. Equation (4) can be used to draw up an ion balance sheet in an easy way. Taking 100 electrons flowing through the electric circuit as the basis for calculation, the number, *N*_*i,100*_, of each ion transported by migration through the solution is:

(5) $$N_{i,100\;}\cdot =\cdot \frac{t_{i}}{z_{i}}100$$

**Fig. 4:**
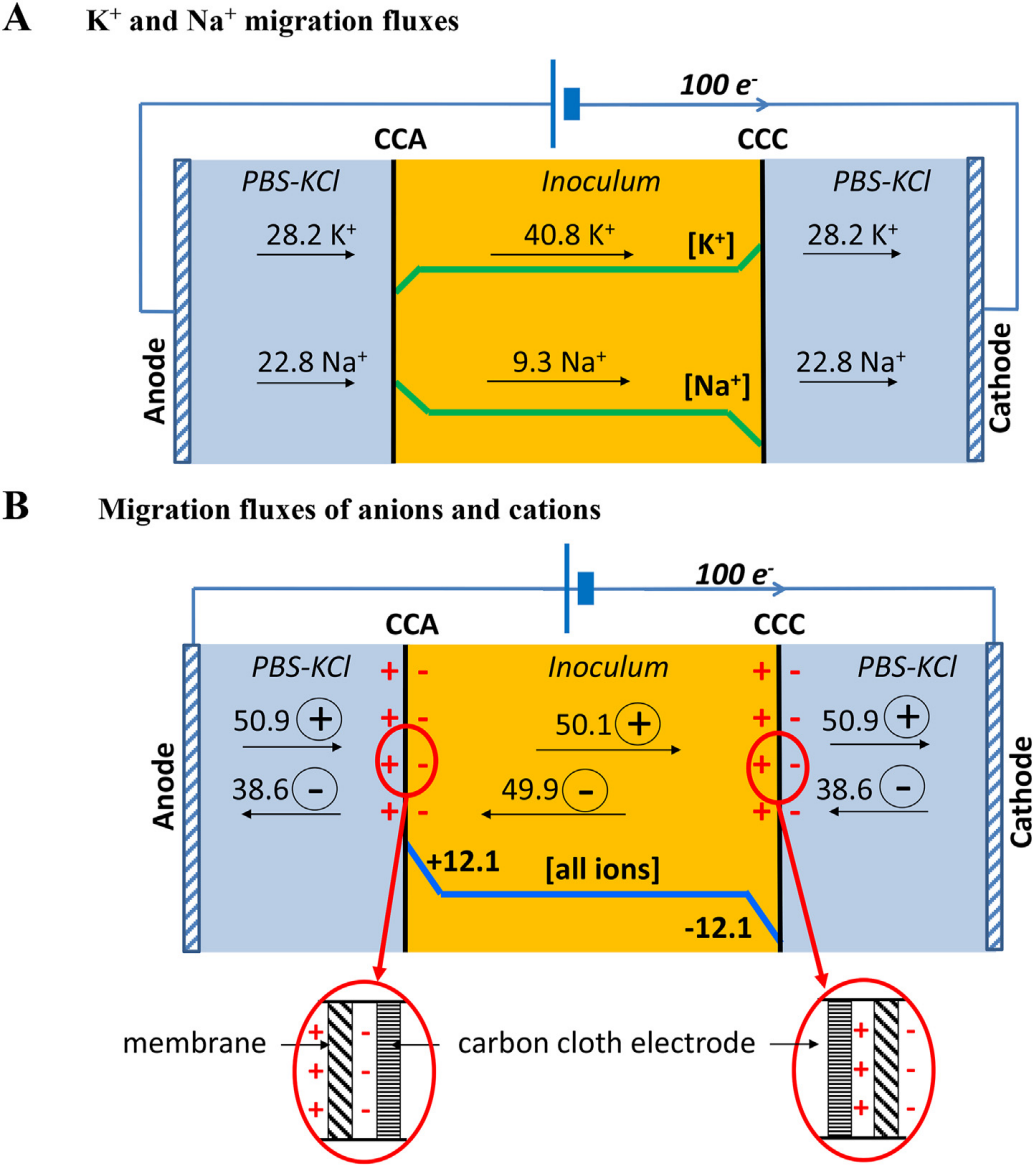
Ion fluxes induced by the electric field at the CCA and CCC interfaces during Step 2. KCl was present in the end compartments.N_i,__100_ are extracted from Table 2. A) The number of K^+^ and Na^+^ ​ions that flowed through the solutions when 100 electrons were flowing through the electric circuit (Ni,100) are reported on the scheme. The electric field induced ion gradients at the interfaces where the electrodes were located, resulting in K^+^ accumulation at CCC and Na^+^ ​accumulation at CCA. B) Values of N_i_,_100_ summed for the cations and the anions. Migration induced a gain of 12.1 ions at the CCA interface and symmetrically a loss of 12.1 ions at the CCC interface. The surface charges, noted in red on the scheme, resulted from the anion and cation directions on each side of the interface. The zooms at the CCA and CCC interfaces recall the accurate position of the CC electrodes with respect to the membrane. (For interpretation of the references to colour in this figure legend, the reader is referred to the Web version of this article.)

The carbon cloth electrodes were positioned against the membranes, at the interface between the inoculum solution (compost leachate) contained in the central compartment and the synthetic medium contained in the end compartments (Fig. 1, Phase 2). The values of *N*_*i,100*_ are reported in Table 2 for the two media. The chemical compositions of the two media were different. The compost leachate contained mainly 60 ​mM KCl and 20 ​mM sodium acetate. Other ions may have been present in small concentrations in the compost leachate but, to obtain a reliable assessment of ion transport in solution, it is sufficient to consider only the ions with the highest concentrations. The synthetic medium of the end compartments was phosphate buffer with or without 60 ​mM KCl added. Migration consequently resulted in different ion fluxes on either side of the interface. Considering K^+^, in the central compartment, migration moved 40.8 K^+^ ions from CCA towards CCC. At the same time, in the end-compartments, when KCl was present, migration moved 28.2 K^+^ ions towards CCA and the same number away from CCC (Fig. 4A). Consequently, K^+^ was depleted by migration in the CCA zone and accumulated in the CCC zone. The situation of the Na^+^ ions was the opposite. Na^+^ accumulated by migration at CCA (9 ions leaving and 23 arriving) and depleted at CCC.

**Table 2:** Transport numbers and ion fluxes. The inoculum solution was assumed to be mainly composed of 60 ​mM KCl and 20 ​mM sodium acetate. The solution in the end compartments was composed of phosphate buffer pH 7, sodium acetate, and addition or absence of 60 ​mM KCl. For each ion species (i), the transport number (ti) was calculated by equation (3) using the values of molar ionic conductivity (λ^0^_i_, 10^-4^ m^2^.S.mol^-1^) from Oliot et a*l.*[52]*.*

	K^+^	Na^+^	Total cations	Cl^-^	CH_3_COO^-^	HPO_4_^2-^	H_2_PO_4_^-^	Total anions
*z*_*i*_	1	1		1	1	2	1	

*λ*_*i*_^*0*^ (10^-4^ ​m^2^.S.mol^-1^)	73.5	50.1		76.3	40.9	114	36	
*Inoculum (central compartment)*								
*C*_*i*_ (mol.m^-3^)	60	20		60	20	0	0	
*t*_*i*_	0.408	0.093		0.424	0.076	0	0	
*N*_*i,100*_	40.8	9.3	50.1	42.4	7.6	0	0	49.9
*PBS with KCl (end compartment)*								
*C*_*i*_ (mol.m^-3^)	86	102		60	20	41	26	
*t*_*i*_	0.282	0.228		0.204	0.036	0.208	0.042	
*N*_*i,100*_	28.2	22.8	50.9	20.4	3.6	10.4	4.2	38.6
*PBS without KCl (end compartment)*								
*C*_*i*_ (mol.m^-3^)	26	102		0	20	41	26	
*t*_*i*_	0.142	0.380		0	0.061	0.348	0.070	
*N*_*i,100*_	14.2	38.0	52.2	0	6.1	17.4	7.0	30.4

Ion fluxes (N_i,100_) were calculated as the number of ions that flowed through the solution when 100 electrons were flowing through the electric circuit, according to Equation (5). Multiplying these values by J_flied_/100.F gives the molar flux densities Φ_i_ in mol.s^-1^.m^-2^.

The global ion fluxes towards and away from the CCA and CCC electrodes can be calculated by summing the molar flux densities of, on the one hand, the cations and, on the other hand, the anions. Always on the basis of 100 electrons flowing through the electric circuit, when KCl was present in the end compartments, Fig. 4B shows that migration brought a gain of 12.1 ions to CCA: 50.9 cations were coming from the end compartment and 49.9 anions from the central compartment (100.8 ions coming at CCA), while only 50.1 cations were pushed away to the central compartment and 38.6 anions to the end compartment (88.7 ions away from CCA). Symmetrically, CCC underwent a loss of 12 ions. The situation was similar in the absence of KCl but the number of ions gained by CCA and lost by CCC was 22 ions for 100 electrons. Migration caused an imbalance in the ionic fluxes at the interfaces where the carbon cloth electrodes were located. The ionic concentration increased at CCA while it decreased at CCC.

These interfacial gradients were balanced by diffusion and by electro-osmosis, until a stationary state controlled by the rate of the different processes is achieved. We are considering only the imbalance provoked by migration here because the diffusion, which took place even when no electric field was applied during the Step 2, did not lead to colonisation of the CCA and CCC electrodes.

**Fig. 5:**
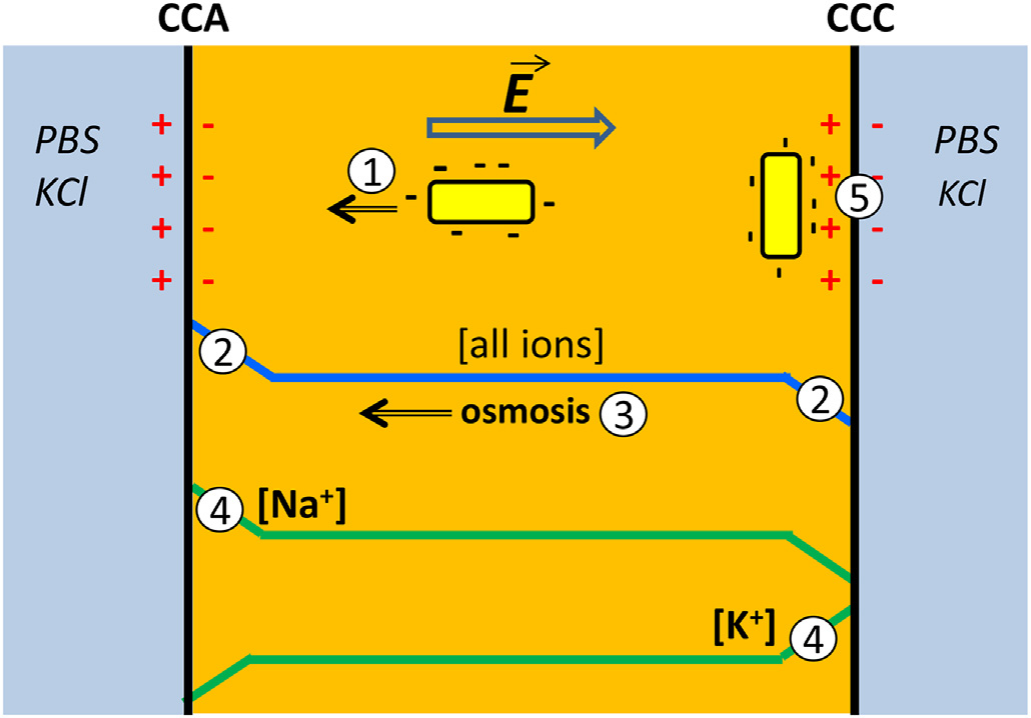
Possible mechanisms for bacteria to approach a surface under an electric field. Microbial cells can be driven to CCA by migration (1) due to their negative surface charge and by the electro-osmosis (3) induced by the interfacial osmolarity gradient (2). Both processes should favour the approach to CCA electrodes only. In fact, the contrary was observed: CCC and CCA electrodes were colonized similarly. To explain this similar colonisation, here the hypothesis is proposed that electroactive bacteria reach the CCA and CCC surfaces by detecting the interfacial gradients of Na^+^ and K^+^, respectively (4). The positively charged surface, which was induced at the side of the CCC electrodes facing the inoculum, favoured cell anchorage (5) and can explain the slightly faster electroactive biofilm formation on the CCC electrodes. This mechanism is not a long-range process that can drive the far approach of bacterial cells but essentially impacts the short-range cell-surface interaction.

The gradient of osmolarity resulting from the migration fluxes provoked so-called electro-osmosis, i.e. water molecules moved towards CCA, where osmolarity was higher than in the bulk and, in contrast, moved away from CCC (Fig. 5). This global motion of water molecules can lead to the transport of microbial cells as already reported [53]. Nevertheless, once again, this phenomenon could explain the colonisation of the CCA electrodes, but it cannot explain the microbial colonisation of CCC observed here, because the electro-osmosis flux moved away from the CCC electrodes.

To sum up, electroactive biofilm formation was enhanced by the electric field applied during electrode exposure to the inoculum. Some phenomena, such as migration and electro-osmosis, might be evoked to explain this effect on the CCA electrodes, but the effect on the CCC electrodes, which attracted the same microbial species, cannot be explained by these assumptions. None of the hypotheses evoked so far can explain how the electric field could lead microbial electroactive species to the CCC electrodes. In contrast, the effect of the electric field on the CCC electrodes can be explained by assuming that microbial cells have the capacity to detect the interfacial ionic gradient of K^+^ created by the electric field.

The capacity of bacterial cells to sense osmolarity was reported long ago [[54], [55], [56]]. It has been shown recently that bacterial cells can establish cell-to-cell communication [57] and long-range electrical signal transmission [58] by detecting K^+^ concentration fields. The electrical signal transmitted by K^+^ concentration gradient has been shown to affect bacterial motility [59]. The K^+^ concentration field created by an existing biofilm can thus attract distant cells towards the biofilm. Furthermore, this long-range communication system has been postulated to be species independent, thus enabling cross-species interactions [59]. It has recently been shown that potassium ion channels play a key role in one of the most efficient electroactive species, *Geobacter sulfurreducens*. Blocking its K^+^ channels inhibited its capability to form electroactive biofilms [60] Here, *G. sulfurreducens* was one of the dominant species in all biofilms.

The capacity of bacterial cells to detect ion concentration gradients can explain the enhancement effect of the electric field on electrode colonisation, which was observed here. The electric field created an ionic gradient at the interface where the electrodes were located. Here, K^+^ was accumulated at the CCC electrode and Na^+^ at the CCA electrode. Bacteria can consequently be attracted by the local K^+^ gradient created at the CCC electrode in the same way as they have been shown to be attracted by a K^+^ concentration gradient created by an existing biofilm [59].

Both CCC and CCA electrodes displayed similar microbial colonisation, as shown by epifluorescence imaging, and similar microbial communities, as shown by 16S rRNA gene sequencing. It must be concluded that similar processes were the motor of colonisation of the surfaces of CCA and CCC electrodes. The increased concentration of K^+^ at the CCC electrode can explain why the cells reached its surface. In contrast, at the CCA electrodes, K^+^ was depleted, but Na^+^ was accumulated. This suggests that electroactive cells should also be able to detect Na^+^ gradients and use them to orient their motion. It has already been demonstrated with K^+^ gradients for several bacterial species, including *G. sulfurreducens*. The existence and similarity of voltage-gated K^+^ and Na^+^ channels on cell membranes support the hypothesis that both Na+ and K^+^ interfacial gradients are used by electroactive cells to reach surfaces [61].

### 3.5 Surface charge distribution

Finally, the ionic balance at the CCA and CCC interfaces can also be considered in terms of charge fluxes. At the CCC interface, the side of the membrane exposed to the inoculum solution saw the cations arrive and the anions move away (Fig. 4B). This pattern favoured a local positive charge of this side of the interface. Conversely, the side of the membrane turned to the end compartment, saw the anions arrive and the cations move away, favouring a negative surface charge on this side. Obviously, the global charge of the interface remained equal to zero as the ion fluxes induced only a charge dissymmetry between the two sides of the interface by promoting a positive surface charge on the face exposed to the inoculum. The CCC electrodes were located in the area where there was an excess of positive charges (Fig. 4B). Symmetrically, the similar dissymmetry promoted a negative surface at the CCA electrodes.

It is known that positively charged surfaces promote the adhesion of microbial cells [62]. Here, the positive surface charge on the CCC electrodes can explain the capacity of the CCC electrodes to establish electroactive biofilms faster than the CCA electrodes. Nevertheless, it must be noted that the surface charge can only act over a short range, which can affect cell adhesion and the start of electroactive biofilm establishment, but not the motion of free cells towards the surface. It acts on cell attachment and early biofilm development, but not on long-range cell approach.

To sum up, both the CCA and CCC electrodes showed the same general behaviour because the interfacial ion gradients were the long-range motor of the cell motion, from the inoculum to the electrode. The slight difference in starting times between CCA and CCC electrodes can be explained by the short-range action due to the surface charge status of electrodes.

## 4 Conclusions

According to the results described here, the ion concentration gradient of K^+^ and Na^+^ created by an electric field at a solid surface can be detected by bacterial cells and used to reach the surface. Here, the interfacial ion gradients resulted from a specific experimental set-up that allowed two different solutions to be separated. Such a set-up was necessary to distinguish the influence of the electric field from that of electrode polarisation. Nevertheless, the same kind of ionic flux is created at the surface of any polarised electrode that supports an electrochemical reaction. Therefore ion gradient at material surfaces should now be considered as a key factor of the long-range detection of electrodes by bacterial cells. As this phenomenon addresses the preliminary phase of biofilm formation, the cell approach phase, it may offer powerful ways to act on, boost, or mitigate the biofilm, or guide it towards a desired state. This would be of particular interest for technological purposes related to both the virtuous side, microbial electrochemical technologies, and the pernicious side, microbial corrosion, of electroactive biofilms.

Considering the ubiquitous presence of endogenous electric fields in living organisms [30,31] and the huge number of interfaces between the different tissues that compose them, the results described here may also impact biomedical research. Similarly to what was achieved in the present experimental set-up, in living organisms, the interfaces between different tissues separate media with different ionic compositions. Endogenous electric fields can consequently create interfacial ion gradients at these interfaces, as observed here. The hosted bacteria may be able to use these ion gradients to detect interfaces, e.g. organ surfaces, and form biofilms on them. This may happen on the natural interfaces that exist between different tissues and also on the artificial interfaces created by implanted materials. The extraordinary capacity of bacteria to detect and infect the surface of prostheses [63], so fast after implanting, is an example for which the ability of bacteria to detect interfacial ionic gradients to move towards surfaces should be considered. Moreover, the recent discoveries of the presence of electroactive microorganisms in living organisms [64,65] warrant the promotion of bacterial electroactivity as a promising field of investigation in the biomedical domain.

## CRediT authorship contribution statement

**Poehere Chong:** Investigation, Formal analysis, Writing – original draft, Visualization. **Benjamin Erable:** Conceptualization, Methodology, Validation, Formal analysis, Supervision. **Alain Bergel:** Conceptualization, Methodology, Validation, Formal analysis, Writing – original draft, Writing – review & editing, Visualization, Supervision, Funding acquisition.

## Declaration of competing interest

The authors declare that they have no known competing financial interests or personal relationships that could have appeared to influence the work reported in this paper.
