# Supplementary material for: How bacteria use electric fields to reach surfaces
Source: Biofilm. 2021 Apr 8;3:100048. doi: 10.1016/j.bioflm.2021.100048 (PMC8090995; doi:10.1016/j.bioflm.2021.100048)
Supplement: Multimedia component 1 [file mmc1.pdf]

How bacteria use electric fields to reach surfaces

Poehere Chong, Benjamin Erable, Alain Bergel*
Laboratoire de Génie Chimique, Université de Toulouse, CNRS, INP, UPS, Toulouse, France
**Corresponding author:* alain.bergel@ensiacet.fr

Supplementary data

**Composition of the macronutrients solution**	
NH_4Cl	28g/L
$MgSO_4$ x $7H_2O$	10g/L
$CaCl_2$ x $2H_2O$	0,57g/L
**Composition of the micronutrients solution**	
$FeCl_2$ x 4H2O	2g/L
$CoCl_2$ x $6H_2O$	1g/L
$MnCl_2$ x $4H_2O$	0,5g/L
$ZnCl_2$ x $2H_2O$	0,05g/L
H_3BO_3	0,05g/L
$CuCl_2$ x $2H_2O$	0,04 g/L
$(NH_4)_6Mo_7O_{24}$ x $4H_2O$	0,07g/L
$NiCl_2$ x $6H_2O$	1g/L
Na_2SeO_3 x $5H_2O$	0,16g/l
HCl 37%	2mL/L
**Composition of the vitamins solution**	
Pyridoxine HCl	1g/L
Nicotinic acid	0,5g/L
Riboflavin	0,25g/L
Thiamin HCl	0,25g/L
Biotin	0,2g/L
Folic acid	0,2g/L
Cobalamin	0,01g/L

Table S.1. Chemical composition of the macronutrient, micronutrient and vitamin solutions.

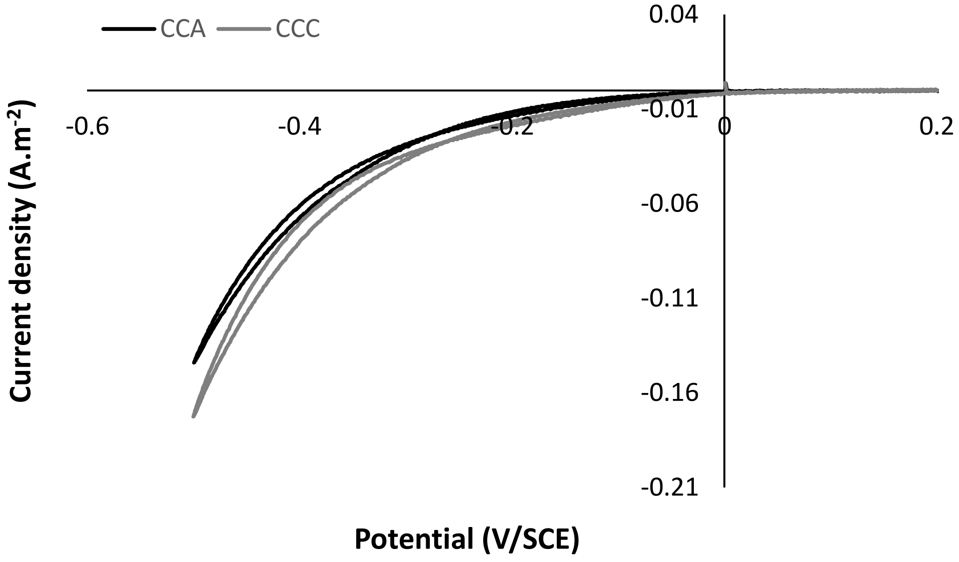

Figure S1. Cyclic voltammetry performed on CCA and CCC electrodes at the end of Step 1. The pre-treatment was considered effective when the two records were similar.

CURRENT RECORDS DURING STEP 3

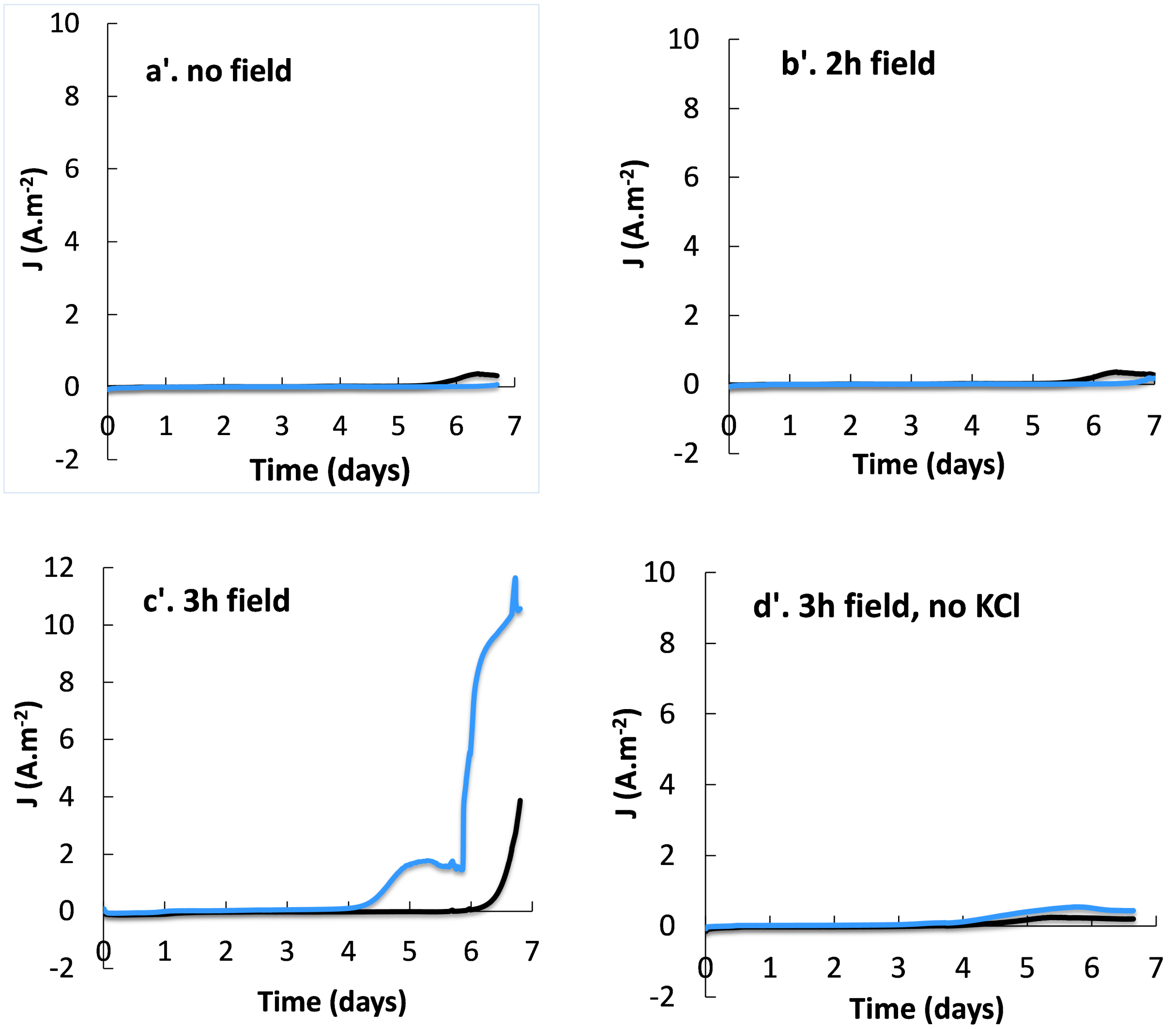

VOLTAMMETRIES AT THE END OF STEP 3

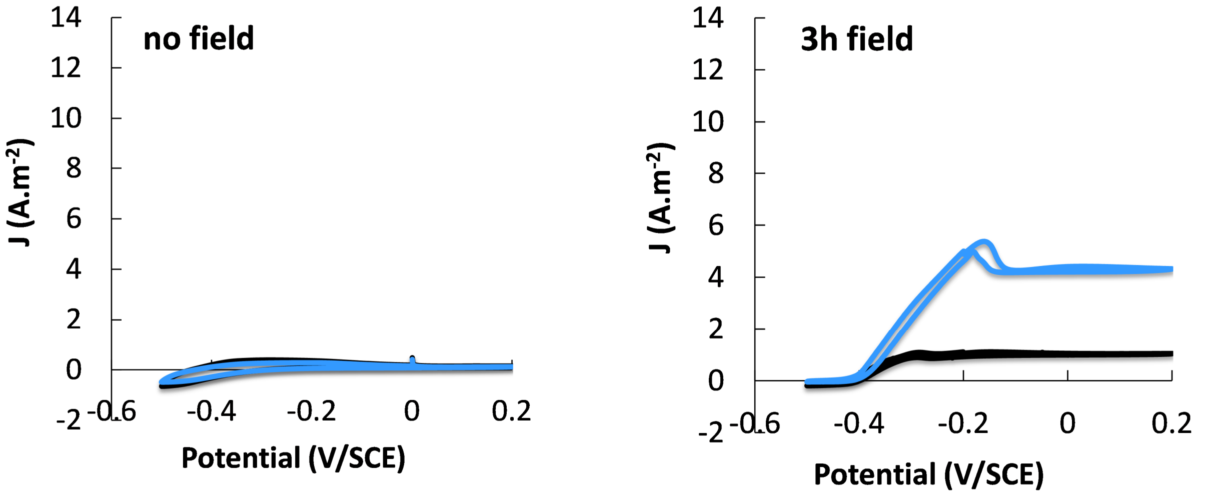

Figure S2. Currents recorded during the 7-day biofilm development and cyclic voltammetries performed on the 7-day biofilms (Step 3). Replicates of the experiments reported in Figure 2. During Step 3, the CCC (blue lines) and CCA (black lines) electrodes were identically polarised at -0.20 V vs. SCE for 7 days, while the current was recorded. The production of current reflected the development of an electroactive biofilm on the electrode surface. The current resulted from the oxidation of acetate to CO_2, which was possible only in the presence of a biofilm that catalysed the reaction. No current was produced when no electric field had been applied during Step 2 (records a'). A small current density was obtained when the electric field was applied for only 2 hours in Step 2 (records b'). In this case, the current density obtained after 2h electric field was similar to that obtained in the control experiments performed without electric field. In contrast, considerable current density was observed when the electric field was applied for 3 hours (records c' and d'), with lower values when there was no KCl in the end compartment (records d').

Cyclic voltammetry performed at the end of the 7-day polarisation period confirmed the common characteristic of an electroactive biofilm when the 3-hour electric field had been applied, while no signal was displayed when the electric field had not been applied. The formation of an electroactive biofilm during Step 3 required the presence of the electric field during exposure of the electrodes to the inoculum (Step 2). When the 3-hour electric field was applied, CCC electrodes started to produce current sooner than CCA electrodes and/or produced higher current density.

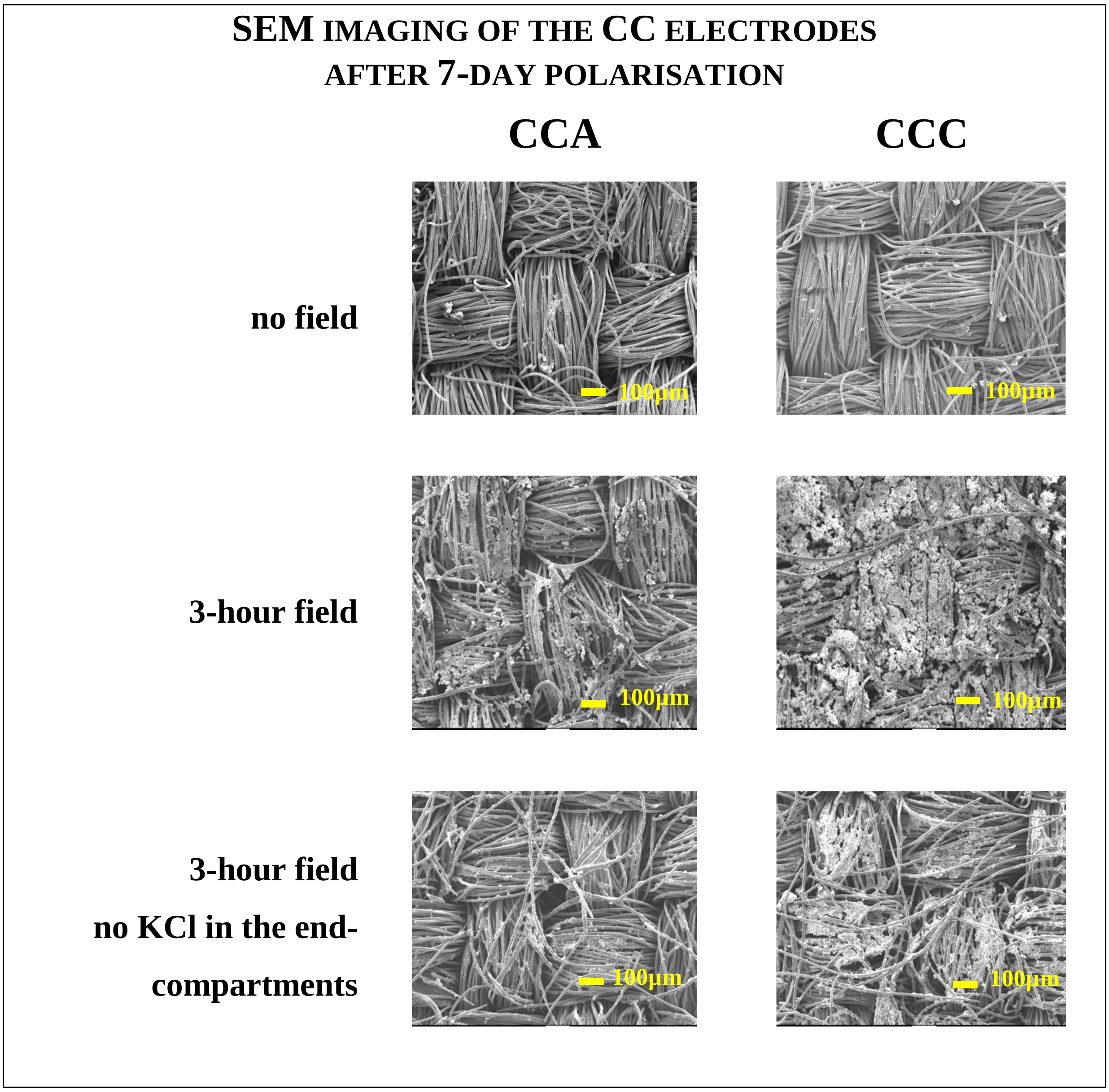

Figure S3. Scanning electron microscopy (SEM) images of the carbon cloth electrodes (CCC and CCA) obtained after 7 days of polarisation at -0.20 V/SCE. SEM showed almost clean surfaces when no electric field had been applied during Step 2. When the electric field was applied for 3 hours, only scattered bacterial colonies and or deposits were detected on CCA electrodes, and a visible coating was observed on CCC electrodes, which was more important when KCl was present in the end compartments. At higher magnification, microbial aggregates and a few salt crystals were observed on the CCA electrodes, while CCC electrodes exhibited noticeable salt precipitation. The electric field consequently promoted the formation of deposits on the surface of the electrodes more pronounced on the CCC electrode.

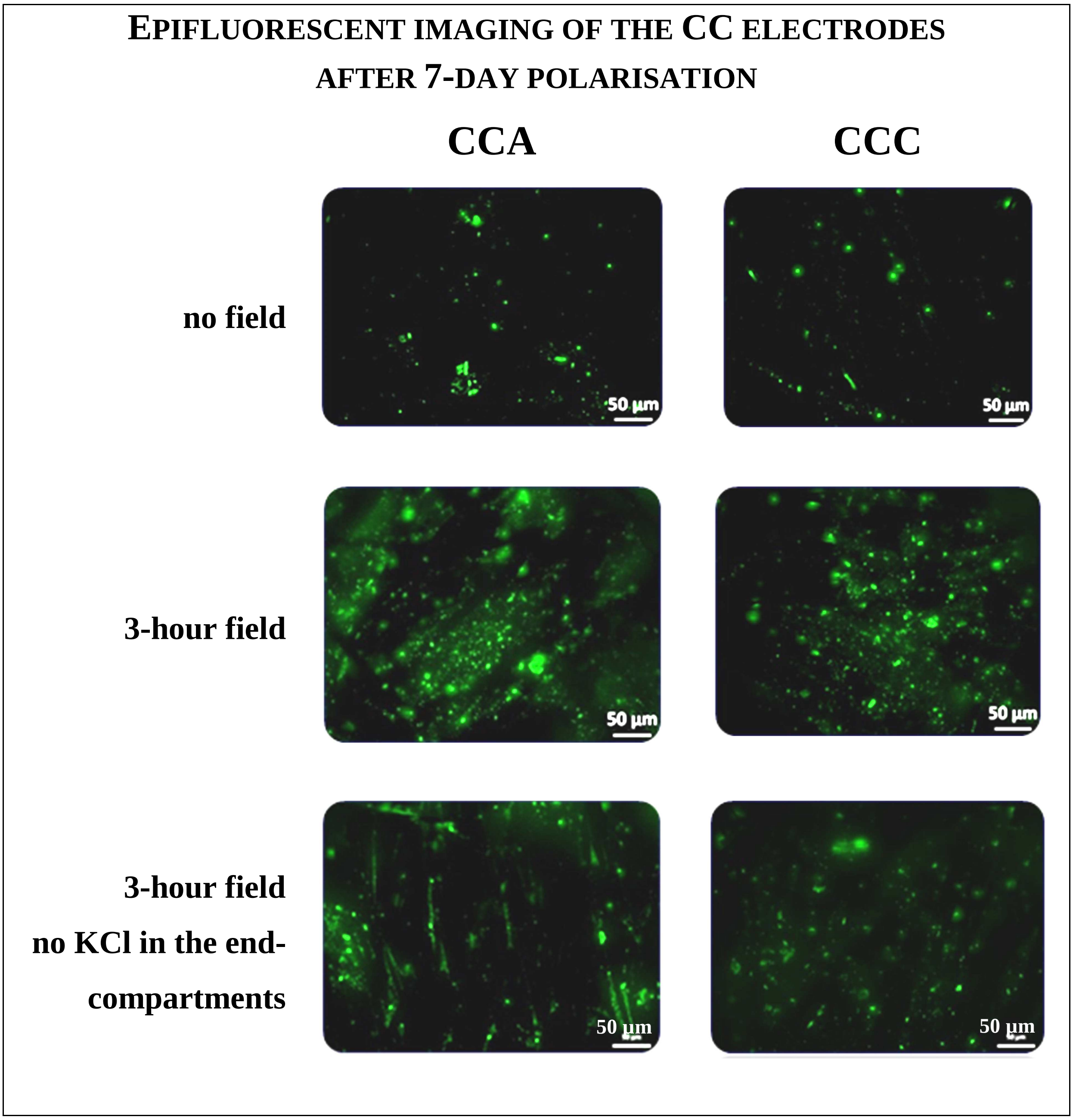

Figure S4. Epifluorescence images of the carbon cloth electrodes (CCC and CCA) obtained after 7 days of polarisation at -0.20 V/SCE. Epifluorescence imaging showed that only a few isolated spots of the electrode surfaces were colonised when no electric field had been applied during Step 2. In contrast, when the electric field had been applied, significant microbial colonisation was observed on both CCC and CCA electrodes, with no apparent difference. On both CCA and CCC electrodes microbial colonisation was more marked when KCl was present in the end compartments. Microbial colonisation was clearly enhanced by the application of the electric field during Step 2, in similar ways on the CCA and CCC electrodes.
